# Human visual perception‐based grayscale contrast enhancement with adaptive window settings in computed tomography images

**DOI:** 10.1002/acm2.70212

**Published:** 2025-09-25

**Authors:** Wei Zhou, Yi Tian

**Affiliations:** ^1^ Department of Computed Tomography (CT) Concept Siemens Shanghai Medical Equipment Ltd. Shanghai China

**Keywords:** CT image quality, display windowing, grayscale contrast enhancement, human visual perception

## Abstract

**Background:**

Grayscale contrast plays a significant role in medical imaging. This is due to the fact that visual examination, where images are represented with different gray shades on display devices, is essential in current medical applications.

**Purpose:**

The effective contrast enhancement should have the ability to provide sufficient grayscale contrast and natural image appearance in line with human visual perception (HVP). To achieve the goal, the paper proposes a novel method for grayscale contrast improvement in computed tomography (CT) diagnostics.

**Methods:**

A new quantitative physical metric incorporating certain human visual characteristics, termed as contrast‐perceived to spatial frequency ratio (CPSFR), is presented to assess the perceptual quality of grayscale‐based images. The window settings achieved by maximizing CPSFR, *Window Settings Automatic*, was evaluated and compared with *Window Settings Preset* and *Window Settings Manual* both of which are most used in CT diagnostics in terms of target detectability and diagnostic satisfaction. Experiments were carried out with 720 phantom images and 80 patient images. In phantom study, images were acquired with the routine body protocol at varying dose levels and assessed by four CT physicists for identifying signal‐present or signal‐absent. In clinical study, images with liver lesions were evaluated and rated by three radiologists with a 4‐point diagnostic quality score.

**Results:**

The phantom study indicated a statistically significant improvement with *Window Settings Automatic*, as compared to *Window Settings Preset* and *Window Settings Manual* (all *p <* 0.01) in accuracy and sensitivity for consensus readings. And the clinical study demonstrated *Window Settings Automatic* had the distinct advantage over other candidates (highest mean score 3.0; *>* 40% of all top‐scoring votes).

**Conclusions:**

The proposed method is capable of enhancing grayscale contrast in the region that really interests observers and yielding natural image appearance well suited to human visual perception to improve target detectability and diagnostic performance.

## 1 INTRODUCTION

A considerable amount of research has driven noteworthy contrast enhancements for a variety of medical images.^1^, ^2^, ^3^, ^4^, ^5^, ^6^ However, they still have the following intrinsic limitations.

### 1.1 Human visual characteristics excluded

In current medical diagnostics, human eyes are the ultimate receiver for medical images. Images should be presented in line with the visual characteristics for assessing and interpreting by human observers.

Many research efforts have been made toward developing visual perception‐based quality assessment^7^, ^8^, ^9^ and exploiting human eyes characteristics for image contrast enhancement.^10^, ^11^ However, to our knowledge, none of these methods have been applied in clinical practice.

### 1.2 Linear mapping relationship unsustained

The drawback common to existing contrast enhancement methods is that the resultant images look far from natural appearance and the extend of enhancement is not controllable well. After the enhancement, the linear mapping, for some or all pixels, between the intensity value and the grayscale level may alter. It is likely for a processed image to have a less natural appearance, which may not be acceptable to the radiologist who have been used to conventional windowing technique,^12^ that is, performing a linear mapping of input intensity values to output grayscale levels to highlight structures of interest by manipulating the display window settings consisting of window width (*WW*) and window level (*WL*) for CT images.

One criticism of intensity windowing is that it does not solve the common issue—when visualizing images their dynamic range can be much wider than the typical display dynamic range.

A few comparisons^13^, ^14^, ^15^, ^16^ have been made between intensity windowing and some contrast enhancement techniques which can display the full dynamic intensity range of a CT image. However, these methods have no significant advantage over conventional window settings in terms of diagnostic performance.

On the other hand, the use of organ‐ or tissue‐specific windows for CT interpretation are increasingly believed as the way to improve diagnostic accuracy. Some specialized window settings have been reported for CT diagnostics, which have helped in abnormalities detection,^17^, ^18^, ^19^, ^20^ nodule size measurement^21^ solid components evaluation^22^ and virtual monoenergetic imaging.^23^, ^24^, ^25^ It is time‐consuming or even impractical for each clinical case to set up its own window manually, but the studies reveal that the diagnostic performance could be improved if the appropriate display window is achieved to present adequate details about the region of interest to radiologists, even with limited dynamic intensity range.

The purpose of this study was to assess the value of a new image quality assessment metric incorporating visual properties for achieving the enhanced and natural image appearance suited to human observer to improve target detectability and diagnostic performance.

## 2 METHODS

### 2.1 Algorithm

In CT diagnostics, the observer visually evaluates an image that is converted by window settings from original CT numbers into different gray shades with the following equation

(1) $$GS\left(x,y\right)=\left\{\begin{array}{c} 0,CT\left(x,y\right)\leq WL-\frac{WW}{2}\\ \frac{CT\left(x,y\right)-\left(WL\right.-\left.\frac{WW}{2}\right)}{WW}*GS_{max},WL\\ -\frac{WW}{2}<CT\left(x,y\right)\leq WL+\frac{WW}{2}\\ GS_{max},CT\left(x,y\right)>WL+\frac{WW}{2} \end{array}\right.\;,$$

where CT(x,y) is the CT number of the image pixel (x,y) and GS(x,y) is its corresponding grayscale level. WW and WL are window width and window level respectively. $GS_{max}$ denotes the maximum gray shade represented by the display device.

CT values above the window will be displayed as white and values below the window as black, and the original CT values within the window is mapped linearly to the full grayscale of the display device.

Hence the image contrast presented is actually not the CT number difference, but the grayscale level difference defined as below equation

(2) $$Contrast^{displayed}=\left|GS_{bg}-GS_{obj}\right|\;,$$

where $GS_{obj}$ and $GS_{bg}$, converted by (1), are the average grayscale levels of a defined targeted region of interest and the background region surrounding the object respectively.

Quality of imaging systems for diagnostics is identified by human visual perception (HVP). The sensitivity of visual perception to grayscale difference depends primarily on two factors from image visualization point of view. One is the average background luminance behind the targeted pixels, and the other is the spatial uniformity of the background luminance.

#### 2.1.1 Background luminance effect

Human eyes have different ability in observing different grayscale. For example, if gray scale darker or lighter, the ability to distinguish between adjacent grayscales is relatively low. However, in the middle position of gray scale, the visual perception is more sensitive to the background brightness difference. Chou, and so on investigated the relationship between the background brightness and the visibility threshold V (also known as the just noticeable grayscale difference) based on subjective experiments^26^ which is given by the following approximate expression

(3) $$V=\left\{\begin{array}{c} T_{0}*\left(1-\sqrt{\frac{GS_{bg}}{127}}\right)+\delta ,0\leq GS_{bg}\leq 127\\ \gamma *\left(GS_{bg}-127\right)+\delta ,127<GS_{bg}\leq 255 \end{array}\right.\;,$$

where $T_{0}$ and γ denote, respectively, the visual threshold when the background grayscale level is 0 and the approximated slope of the line that models the relationship corresponding to higher background luminance (over 127). And δ is the minimum threshold. $T_{0}$, γ and δ are found to be 17, 3/128 and 3, respectively. The maximum visibility threshold, that is, $V_{max}$, is given by the following equation

(4) $$V_{max}=T_{0}+\delta .$$

#### 2.1.2 Spatial masking

It was found that the reduction of the visual sensitivity to contrast is caused by the increase in the spatial non‐uniformity of the background luminance, which is known as spatial masking.

For the M∗N image region, its spatial frequency reflecting the grayscale spatial variation inside is defined as the following equation

(5) $$SF\;\left(M,N\right)=\sqrt{SF_{H}{\left(M,N\right)}^{2}+SF_{V}{\left(M,N\right)}^{2}}\;,$$

where $SF_{H}\left(M,N\right)$ denotes horizontal spatial frequency determined as

(6) $$SF_{H}\left(M,N\right)=\sqrt{\frac{1}{M*\left(N-1\right)}\sum_{x=1}^{M}\sum_{y=2}^{N}{\left[GS\left(x,y\right)-GS\left(x,y-1\right)\right]}^{2}}\;,$$

and $SF_{V}\left(M,N\right)$ is vertical spatial frequency determined as

(7) $$\begin{array}{cc} & SF_{V}\left(M,N\right)\\ & =\sqrt{\frac{1}{\left(M-1\right)*N}\sum_{x=2}^{M}\sum_{y=1}^{N}{\left[GS\left(x,y\right)-GS\left(x-1,y\right)\right]}^{2}}\;. \end{array}$$

#### 2.1.3 Contrast‐perceived to spatial frequency ratio (CPSFR)

The paper takes into account HVP and proposes a new physical assessment metric, namely the contrast‐perceived to spatial frequency ratio (CPSFR), for grayscale‐based image quality.

The image contrast is firstly extended from $Contrast^{displayed}$ to $Contrast^{perceived}$ as the following expression

(8) $$Contrast^{perceived}=Contrast^{displayed}\;*f\left(V\right),$$

where $Contrast^{displayed}$ is defined in (2), and the function f(V) is defined as the following expressions to normalize the visibility threshold V,

(9) $$f\left(V\right)=1-V^{normalized},$$

(10) $$V^{normalized}=\frac{V}{V_{max}}\;,$$

where V and $V_{max}$ are obtained according to (3) and (4) respectively.

And we present the new metric by combining the $Contrast^{perceived}$ and $SF_{bg}$ as the following expression

(11) $$CPSFR=\frac{Contrast^{perceived}}{SF_{bg}}\;,$$

where $SF_{bg}$ denotes the spatial frequency of the background region and is calculated by (5).

**FIGURE 1:**
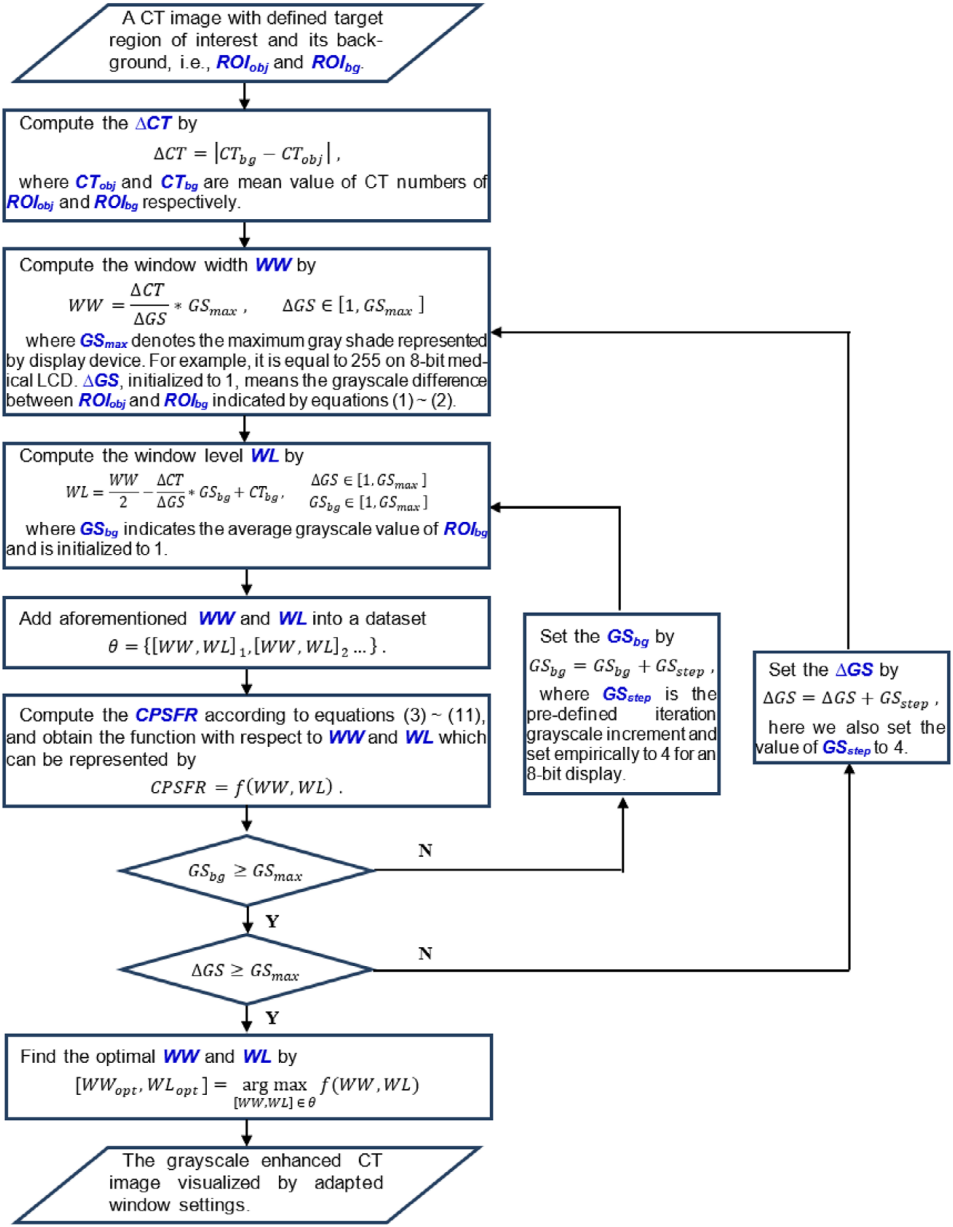
The flowchart of optimizing display window settings by maximizing contrast‐perceived to spatial frequency ratio (CPSFR).

By maximizing CPSFR, we can achieve the optimal window settings with which the image is visualized to conform with HVP. The procedures for implementing are presented in Figure 1.

### 2.2 Experimental setup

#### 2.2.1 Perceptual quality enhancement

We evaluated the method's ability to improve visual impression and its effect on image visualization consistency using both phantom and patient sample images.

#### 2.2.2 Target detectability optimization

**FIGURE 2:**
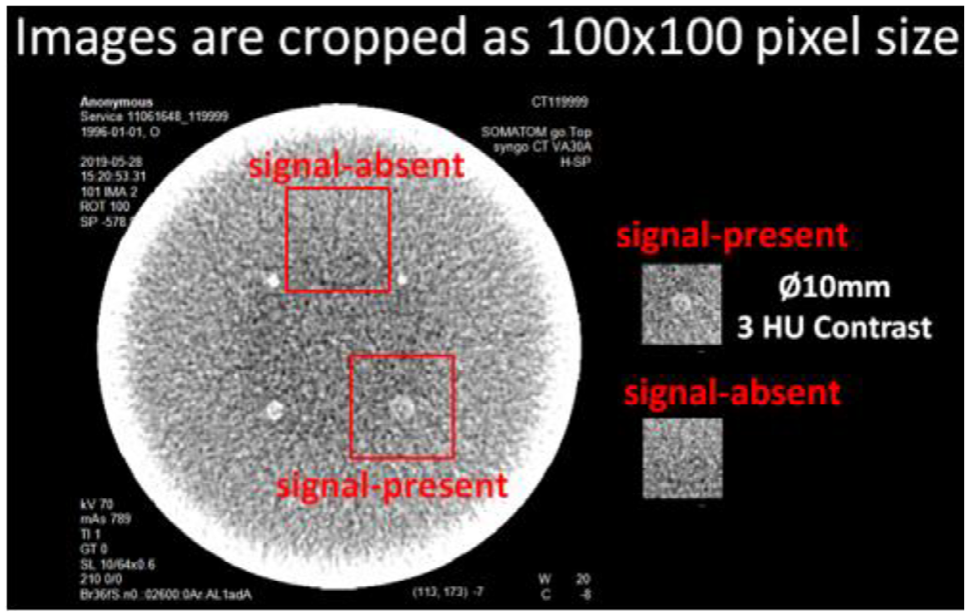
The diagram of samples of signal‐absent and signal‐present (location known). Images were acquired on MITA CT IQ Low Contrast Body Phantom (the Phantom Laboratory) by the Siemens Somatom go.Top scanner (Siemens Heathineers) with the standardized protocol of 64 × 0.6 mm detector configuration and reconstructed with a standard soft tissue kernel algorithm as well as 210 mm FoV. And 10 mm slice thickness is used to mitigate excessive noise in low‐dose test images.

The study introduces a basic signal detection task: the observer receives a stimulus—the phantom image that may or may not contain a weak signal—and must decide whether the signal is present or absent. 3HU contrast with 10 mm diameter rod was selected as the targeting object as Figure 2 shown.

The dataset consisted of 720 images (100 × 100 pixels each), cropped from 36 groups corresponding to the 36 kVp/mAs settings listed in Table 1. Each group contained 20 images acquired from repeated exposures: 10 signal‐present and 10 signal‐absent.

**TABLE 1:** Data was acquired with varying dose levels.

Dose Level	70 kV	80 kV	100 kV	110 kV	120 kV	130 kV
25%	132 mAs	83 mAs	41 mAs	32 mAs	25 mAs	21 mAs
50%	263 mAs	167 mAs	82 mAs	64 mAs	50 mAs	41 mAs
75%	395 mAs	250 mAs	123 mAs	95 mAs	75 mAs	62 mAs
100%	526 mAs	333 mAs	164 mAs	127 mAs	100 mAs	82 mAs
125%	658 mAs	416 mAs	205 mAs	159 mAs	125 mAs	103 mAs
150%	789 mAs	500 mAs	246 mAs	191 mAs	150 mAs	123 mAs

*Note*: 100% dose level equals to 8.8 mGy CTDIvol.

**FIGURE 3:**
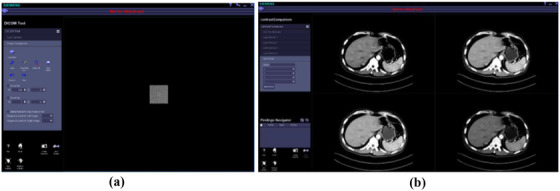
Screenshot of the graphic user interface (GUI) developed in‐house based on MeVisLab (MeVis Medical Solutions AG). The window width and level were hided to the observer, and the image size was not adjustable. (a) is for signal detection tests, which consists of a menu part on the left and a display area on the right. The observer is allowed to view images forward/backward by clicking Previous/Next button, make the decision by picking Yes or No from Dropdowns, and Save results. (b) is for diagnostic satisfaction tests, which consists of a menu part on the left and a display area containing 2 × 2 windows on the right. Each test image, with four different appearances which were achieved using pre‐determined window settings, was presented as four candidates placed randomly in four windows. The GUI enables the observer to view images forward/backward by scrolling the mouse, rate candidates through the Dropdown menu, and Save results. Re‐rating is allowed.

Four physicists, who had respectively 10/8/6/3 years of experience in CT image quality assessment, independently participated in experiments where test images were presented in random order on the interface as shown in Figure 3a for identifying signal‐present or signal‐absent.

Every observer performed three rounds of evaluation test with different sets of window settings as follows,

- *Window Settings Preset*—the window width and level preset. In *Window Settings Preset* round, images were displayed with the fixed *WL* of 40 HU and *WW* of 300 HU, as used in CT body scans.
- *Window Settings Manual*—the window width and level adjusted manually by the observer. In the round, observers were allowed to change the window width and level for each image until his/her desired image impression was achieved.
- *Window Settings Automatic*—the window width and level adapted automatically by the proposed method. Hence each image might have the different window settings. During the evaluation, the window settings were not allowed to adjust by hand.

To avoid visual fatigue, every evaluation round of 720 images was divided into three parts, and only 240 images were viewed per part which took the observer 40–55 min. And there was at least a 3‐day interval between each review part for observers to minimize recall bias.

All the experiments were conducted under low ambient lighting conditions. A 19‐inch liquid crystal display (LCD) monitor for medical with 1920 × 1080 pixels and 8‐bit depth was used, and a constant viewing distance of 40 cm was suggested but not strictly enforced.

#### 2.2.3 Diagnostic performance improvement

A diagnostic satisfaction experiment is introduced to investigate the lesion identification improvement, where the radiologist independently rated test images on an ordinal scale according to his/her visual impression and diagnosis criteria.

The image set consisted of 80 retrospective patient images anonymized which were from 80 clinical cases with malignant liver metastases in enhanced CT scanning. Three radiologists from different institutions with more than 10 years of experience in interpretation and diagnosis of medical X‐ray images participated in the experiments.

Four sets of window settings were pre‐determined as candidates as follows,

- *Window Settings Preset*—the window width and level preset, namely the fixed *WW*/*WL* of 200/40 HU, which are typically used in abdominal CT diagnostics.
- *Window Settings Automatic*—the window width and level resulting from the proposed method applied to each test image, where the targeting object is the lesion, that is, malignant liver tumors.
- *Window Settings Manual 1*—the window width and level that was obtained by manual adjustment of the radiologist 1 according to the diagnostic experience and personal preferences.
- *Window Settings Manual 2*—the window width and level that was determined by the radiologist 2 with the same operating procedures as the radiologist 1.

Each set of window settings consisted of 80 pairs of *WW*/*WL* values, which had the one‐to‐one correspondence with 80 test images. And these 80 items were kept identical in *Window Settings Preset* set, whereas might be different each other in other three window sets since they were determined, manually or automatically, for each image individually.

All four sets of window settings were separately deployed to 80 test images. Thus, the test image could be visualized simultaneously with four different window settings, as shown in Figure 3b. During the experiments, the images were displayed one by one in the same order for each observer.

The radiologists were told that the 4 windows on the screen show the same one image at a time but with different grayscale representations. And it should be noted that two of the three radiologists, namely radiologist 1 and radiologist 2, were the people who determined *Window Settings Manual 1* and *Window Settings Manual 2*, respectively. However, they both did not know that the window settings pre‐determined by themselves had been applied as the candidate.

The experiments were conducted in low ambient light on the same 19‐inch and 8‐bit LCD monitor with 1920 × 1080 pixels. The monitor was positioned approximately 50 cm away from the radiologists, but they were allowed to freely vary the viewing distance. The viewing time was unrestricted, and short breaks were allowed during tests. In practice, the radiologist's viewing times varied from 40 min to an hour.

### 2.3 Analysis

In the phantom image‐based detection task, there are two classes of statistics by which the target detectability was evaluated: the validity, for example, accuracy, sensitivity, and specificity, and the reliability, for example, the rate of inter‐observer agreement. Sensitivity is the proportion of actual true positives identified, specificity is the proportion of actual true negatives identified, and accuracy is calculated by adding hits and correct rejections and dividing by the total number of tested samples. Detection rates of the consensus reading were compared between the proposed *Window Settings Automatic* and each established baseline *Window Settings* (*Preset* and *Manual*) individually. A Bonferroni correction was applied to the significance level to account for the multiple comparisons, and differences with a *p*‐value of less than 0.025 were considered statistically significant.

In the patient image‐based estimation task, the radiologist was ‘forced’ to rate ‘four’ candidates for each image with different diagnostic quality grade which was defined as a qualitative score on a 4‐point scale: 1, dissatisfied; 2, neither satisfied nor dissatisfied; 3, satisfied; and 4, extremely satisfied. It might be not appropriate to treat the numerical ratings in ways that assume the scale is linear. Hence, we investigated the distribution of the highest score of ‘4’.

And a rough speed analysis of the proposed method was performed. All experiments in the paper were conducted based on 8‐bit representation which is sufficient for most CT applications.^27^, ^28^ Therefore, $GS_{max}$ was set to 255 in our algorithm. With MATLAB (Mathworks) running on a HP ZBook of 2.60 GHz CPU and 16 GB RAM, the computation time, averaged over 80 patient images used in the study, was less than 45 ms.

## 3 RESULTS

### 3.1 Perceptual quality enhancement

**FIGURE 4:**
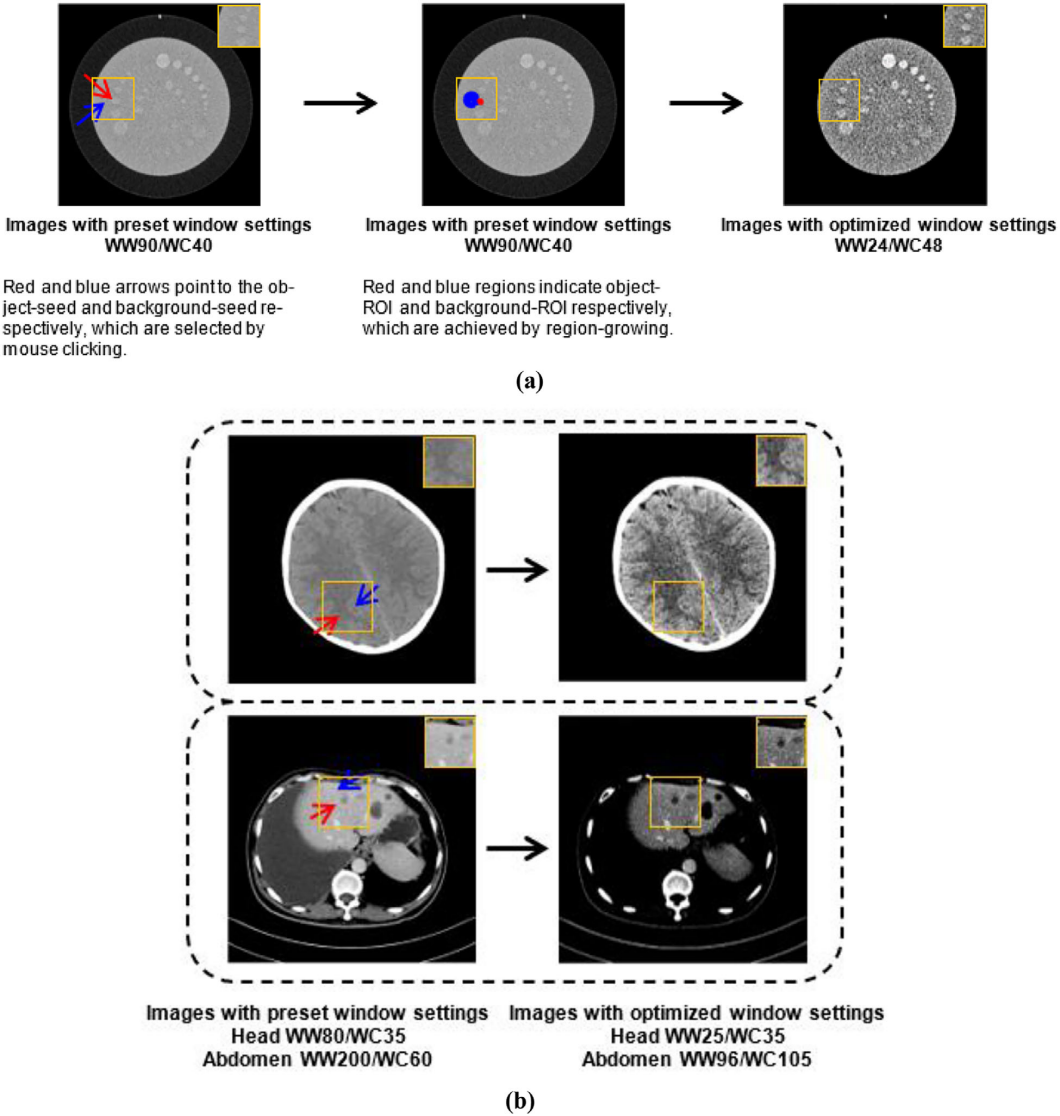
(a) Images with CTP515 module of Catphan500 (The Phantom Laboratory, USA) and (b) clinical images of head and abdomen, respectively, on Siemens Somatom Perspective 128 (Siemens Heathineers, Germany). In (b), the arrows have the same meaning as in (a), and the similarly defined ROIs were not outlined to maintain clarity. The region indicated with the orange box are of interest to the observer in the test.

As shown in Figure 4a and b, the object visibility is improved by representing the targeting object and its background with the grayscale levels which are most effectively distinguished by human eyes.

**FIGURE 5:**
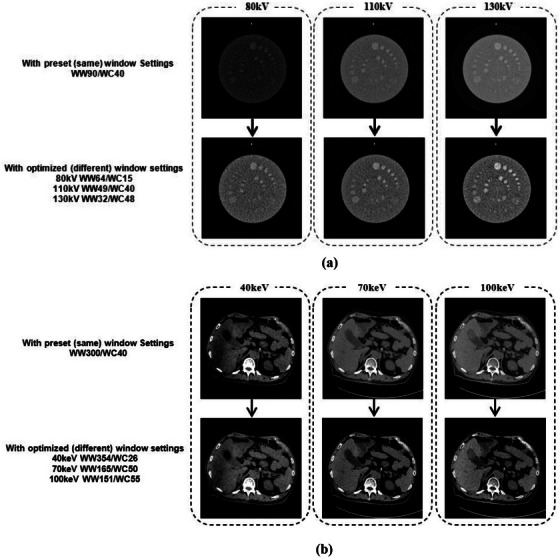
(a) Images of CTP515 module of Catphan500 with different kVs on Siemens Somatom Perspective 128, and (b) the abdomen image at different virtual monoenergetic imaging (VMI) levels with 120 kV acquisition protocol on Siemens Naeotom Alpha (Siemens Heathineers, Germany).

Different scan conditions or reconstruction algorithms can lead to CT number varies due to attenuation differences at distinct energy levels, which results in different image appearance if still the same window settings are used. When images are always presented in accord with HVP, the visual impression is maintained as some level of consistency. Example cases are shown in Figure 5a and b.

### 3.2 Target detectability optimization

As presented in Table 2, the results of the study illustrate that *Window Settings Automatic* has the best detection performance in both validity and reliability aspects, as compared with *Window Settings Preset* and *Window Settings Manual*. It is indicated that the target detectability is enhanced evidently resulting from the window settings optimized by our method, especially in sensitivity and accuracy, not only for each observer but also for inter‐observer agreement.

**TABLE 2:** Overall sensitivity, specificity and accuracy.

	Window settings preset			Window settings manual			Window settings automatic		
Observer	Sensitivity	Specificity	Accuracy	Sensitivity	Specificity	Accuracy	Sensitivity	Specificity	Accuracy
1	33% (120/360)	100% (360/360)	67% (480/720)	65% (234/360)	100% (360/360)	83% (594/720)	78% (282/360)	100% (360/360)	89% (642/720)
2	46% (167/360)	100% (360/360)	73% (527/720)	74% (266/360)	100% (360/360)	87% (626/720)	83% (300/360)	100% (360/360)	92% (660/720)
3	70% (252/360)	98% (351/360)	84% (603/720)	73% (261/360)	100% (360/360)	86% (621/720)	96% (346/360)	100% (360/360)	98% (706/720)
4	86% (311/360)	96% (346/360)	91% (657/720)	80% (287/360)	99% (356/360)	89% (643/720)	94% (337/360)	100% (360/360)	97% (697/720)
Consensus	21% (75/360)	94% (337/360)	57% (412/720)	47% (168/360)	99% (356/360)	73% (524/720)	70% (252/360)	100% (360/360)	85% (612/720)

*Note*: Data inside parentheses were used to calculate percentages.

For consensus readings, the use of *Window Settings Automatic* resulted in statistically significant improvements in both accuracy (*p* < 0.01) and sensitivity (*p* < 0.01) compared to *Window Settings Preset*, as assessed by two‐tailed t tests. Similarly, significant improvements were observed compared to *Window Settings Manual* for both accuracy (*p* < 0.01) and sensitivity (*p* < 0.01).

Differences were not significant (*p* > 0.09, two‐tailed *t* test) between *Window Settings Automatic* and *Window Settings Manual* in the specificity for consensus readings. The high specificity shown in Table 2 reflects the fact that each of four observers has a higher criterion. Unlike radiograph readers who may wish to maximize the number of abnormal cases to avoid missing significant pathology, CT physicists, who are responsible for assessing low contrast detectability of the scanner, certainly are inclined to set a strict criterion to avoid making false alarms. Even so, the *Window Settings Automatic* also outperforms other two window settings at specificity.

### 3.3 Diagnostic performance improvement

**FIGURE 6:**
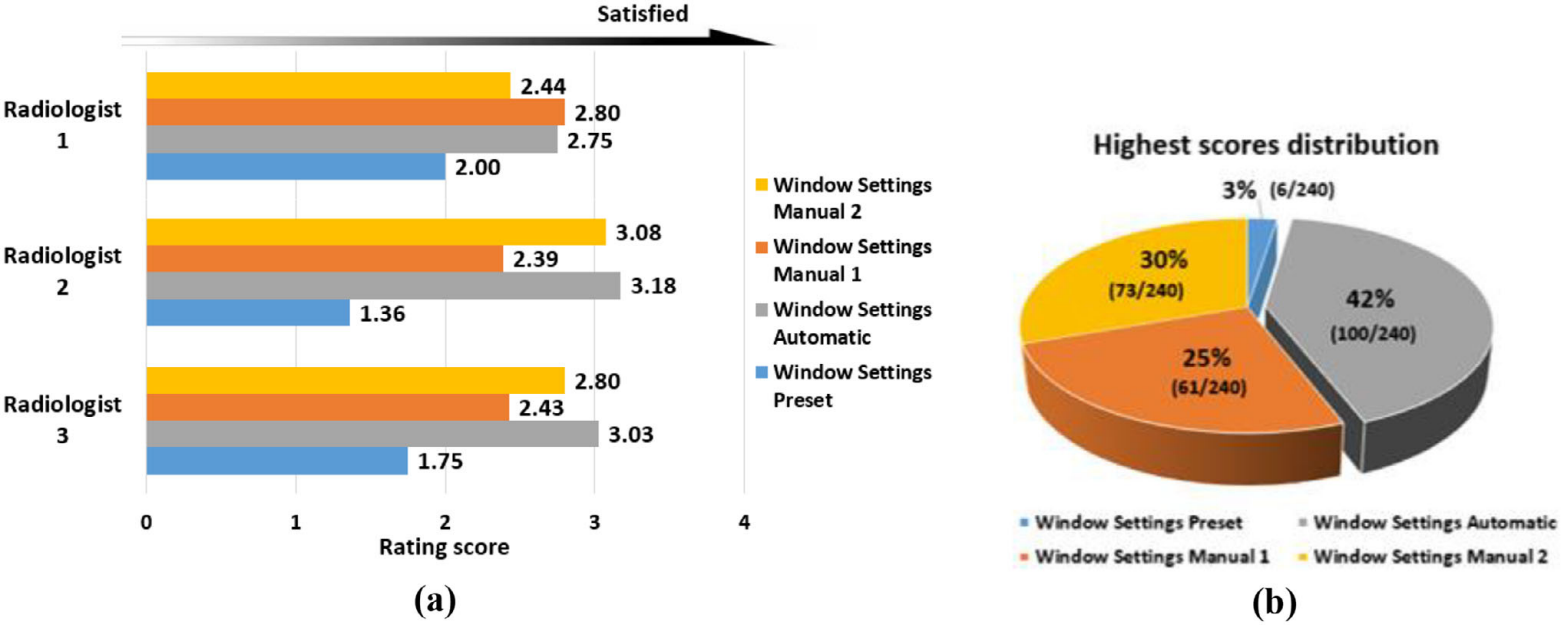
(a) Bar plots are used to illustrate the independent diagnostic satisfaction ratings. (b) The highest score distributions among four sets of display window settings. Data inside parentheses were used to calculate percentages.

The numerical scores, resulting from ratings by three radiologists for 80 testing images with four sets of window settings, are averaged for each window settings set per radiologist, as shown in Figure 6a. And *Window Settings Automatic* has the highest mean score of 2.99 from all radiologists.

The results reveal that *Window Settings Automatic* has the best overall performance among all the candidates. And just to radiologist 1, *Window Settings Automatic* is slightly inferior to *Window Settings Manual 1* which was pre‐determined by radiologist 1 himself. As for radiologist 2, however, *Window Settings Automatic* is superior to *Window Settings Manual 2* pre‐determined by radiologist 2 himself. The radiologist 3, who did not participate in predefining manually the window settings, may be typical of radiologists in common clinical scenarios. And rating results of radiologist 3 show that *Window Settings Automatic* has the distinct advantage over other three window settings.

The results also reflect the fact that individual differences or preferences do exist. For instance, radiologist 2 does not seem to like *Window Settings Manual 1* which is radiologist 1's favorite, and vice versa.

*Window Settings Automatic* wins 42% of all the 240 top‐scoring votes and is greatly ahead of the other three competitors, as shown in Figure 6b.

## 4 DISCUSSION

The paper proposes a new image assessment method, termed as contrast‐perceived to spatial frequency ratio, which includes the visual perception quality. It is a grayscale‐based objective metric and could be complementary to existing intensity‐based ones widely used to evaluate medical image quality. Subsequently an automatic window settings adjustment method is presented to achieve the optimal image appearance suited to human eyes by maximizing the metric. It has the advantage in reducing the work burden and eliminating inter‐ and intra‐observer variability in comparison to conventional window settings adjustment manually.

In essence, our study establishes a new mapping relationship between intensity value and display grayscale for image pixels. Thus, it becomes possible that the method can be extended to a wider range of imaging modalities where the windowing techniques are applied for grayscale mapping, such as chest radiography, mammography, magnetic resonance imaging, and so on.

There are several limitations to our study. First, the further investigation of the way to obtain the region of interest (ROI) might be required. In the present work, an interactive way with observers` mouse clicking on the image is introduced to automatically capture the targeting object and its background. Starting from the selected seed pixel which interests the observer, the well‐known region‐growing is used to generate ROI (illustrated in Figure 4a for example). This process goes on until the so‐called stop conditions are satisfied. But the conditions may have to be tuned separately due to different image characteristics. To define the stop conditions well, it is necessary to carry out experiments involving more clinical data, although we have demonstrated the advantage of the proposed method in enhancing the image visualization, such as for liver, brain, and lung. However, it must be noted that our idea is independent of how ROI is obtained, even with the conventional way of hand‐drawing. Second, there is a lack of assessing the impact of the method on image interpretation time, albeit no significant time difference found between review session with *Window Settings Automatic* and that with *Window Settings Preset* during this study. Third, the number of participants in diagnostic performance experiments may be small, although the results have shown noticeable individual differences. These issues identified in the present study will be addressed in future research.

## 5 CONCLUSIONS

In this paper, we propose a method which is quite effective in enhancing grayscale contrast and improving display quality. CPSFR, a new metric for CT image quality assessment, is developed taking into account the effect of background luminance and spatial frequency on the human visual sensitivity to grayscale differences. The best image impression for human observers is achieved with the adaptive display window settings which are optimized by maximizing CPSFR. Unlike most existing methods for medical image contrast enhancement, the new method enhances the grayscale contrast for local details which the observer really cares about, while retaining the naturalness of the whole image. Experiment results, involving phantom images and patient images, proved that the proposed method has the capability to enhance target detectability and improve diagnostic performance.

## AUTHOR CONTRIBUTIONS

Wei Zhou: Conceptualization, methodology, investigation, formal analysis, validation, literature research, writing—original draft, review & editing, and final approval of the manuscript. Yi Tian: Project administration, manuscript review, and final approval of the manuscript.

## CONFLICT OF INTEREST STATEMENT

The authors have no conflicts of interest to disclose.
